# Selective elimination of breast cancer surgery in exceptional responders: historical perspective and current trials

**DOI:** 10.1186/s13058-016-0684-6

**Published:** 2016-03-08

**Authors:** Raquel F. D. van la Parra, Henry M. Kuerer

**Affiliations:** Department of Surgical Oncology, Netherlands Cancer Institute, Plesmanlaan 121, Amsterdam, 1066 CX The Netherlands; Department of Breast Surgical Oncology, The University of Texas MD Anderson Cancer Center, 1400 Pressler Street, Unit 1434, Houston, TX 77030 USA

## Abstract

With improvements in chemotherapy regimens, targeted therapies, and our fundamental understanding of the relationship of tumor subtype and pathologic complete response (pCR), there has been dramatic improvement in pCR rates in the past decade, especially among triple-negative and human epidermal growth factor receptor 2-positive breast cancers. Rates of pCR in these groups of patients can be in the 60 % range and thus question the paradigm for the necessity of breast and nodal surgery in all cases, particularly when the patient will be receiving adjuvant local therapy with radiotherapy. Current practice for patients who respond well to neoadjuvant chemotherapy (NCT) is often to proceed with the same breast and axillary procedures as would have been offered women who had not received NCT, regardless of the apparent clinical response. Given these high response rates in defined subgroups among exceptional responders it is appropriate to question whether surgery is now a redundant procedure in their overall management. Further, definitive radiation without surgical resection with or without systemic therapy has been proven effective for several other malignant disease sites including some stages of esophageal, anal, laryngeal, prostate, cervical, and lung carcinoma. The main impediments for potential elimination of surgery have been the fact that prior and current standard and functional breast imaging methods are incapable of accurate prediction of residual disease and that integrating percutaneous biopsy of the breast primary and nodes following NCT may circumvent this issue. This article highlights historical attempts at omission of surgery following NCT in an earlier era, the current status of breast and nodal imaging to predict residual carcinoma, and ongoing and planned trials designed to identify appropriate patients who might be selected for clinical trials designed to test the safety of selected elimination of breast cancer surgery in percutaneous image-guided biopsy-proven exceptional responders to NCT.

## Background

A key advantage of neoadjuvant chemotherapy (NCT) is the opportunity to assess response early during treatment as a predictor of pathologic complete response (pCR) at the end of therapy [1, 2]. For the individual patient, achievement of pCR prior to surgery has been shown to confer improvements in long-term disease-free and overall survival outcomes.

The most common definition of pCR includes the absence of invasive disease in the breast and axillary nodes. Prognosis is not affected by the presence of residual in situ disease. The rate of pCR has been shown to vary dramatically depending on the tumor subtype. A large meta-analysis of 11,695 patients in 30 studies revealed pCR to be only 8.3 % for patients with human epidermal growth factor receptor 2 (HER2)-negative/hormone receptor (HR)-positive tumors, rising to 18.7 % for HER2-positive/HR-positive patients, 31.1 % for triple-negative (TN) patients, and 38.9 % for HER2-positive/HR-negative patients [2]. pCR is a strong prognostic marker for superior disease-free and overall survival, especially in the HR-negative groups (either HER2-positive or HER2-negative) as the correlation with survival is best in these groups [3].

With improvements in chemotherapy regimens and targeted therapies according to tumor subtype and nodal status (e.g., trastuzumab and pertuzumab), pCR rates have dramatically improved over recent decades, especially in TN and HER2-positive breast cancer. In these exceptional responders, pCR rates of up to 60 % and higher can be achieved [4, 5]. Current practice for patients who respond well to NCT is often to proceed with the same breast and axillary procedures as would have been offered in women who had not received NCT, regardless of the apparent clinical response. Given these high response rates in defined subgroups among exceptional responders it is appropriate to question whether surgery is now a redundant procedure in their overall management, particularly when patients will often routinely be treated with adjuvant radiotherapy.

## Historical perspective and results of early studies testing omission of surgery based on response to NCT

Prior studies performed in earlier eras before high-quality breast imaging, improved systemic therapy, and our understanding of molecular subtypes and response to systemic therapy seem potentially doomed because clinical response is notoriously inaccurate in determining pathologic residual disease. Radiation with systemic therapy as a definitive local treatment has been proven effective for several other malignant disease sites including some stages of esophageal, anal, laryngeal, prostate, cervical, and lung carcinoma. Early studies presented in Table 1 evaluated radiotherapy as the definitive local modality for treating the breast in patients who have a clinical complete response to NCT, which resulted in unacceptably high locoregional failure rates. Essentially, physical examination and imaging were not able to identify a group of patients without or with only minimal residual disease.

**Table 1:** Overview of studies that compared surgery with radiotherapy alone after neoadjuvant chemotherapy

Study	Study period	*n*	cCR	Locoregional treatment		5-year overall survival		5-year LRR	
				Surgery	RT alone	Surgery (%)	RT alone (%)	Surgery (%)	RT alone (%)
De Lena et al. [11]	1975–1980 prospective	132 T3b-4 N0-2	100 % RT group; 60 % surgery group	65	67	49.1^a^	51.7^a^	29.6	31.1
Perloff et al. [9]	1978–1983 prospective	87	18 %	43	44	63^b^	50^b^	19	27
Scholl et al. [8]	1986–1990	200	?	36 Mtx ± RT, 62 BCS + RT	102	–	–	24	
Touboul et al. [6]	1982–1990 prospective	97	33	37 rD (>3 cm), Mtx; 27 rD (<3 cm), BCS	33	83.3	75.7	16 after BCS, 5.4 after Mtx	16
Ellis et al. [12]	1985–1994	185	39	120; 29 Mtx, 91 BCS	39	76	84	7	21
Mauriac et al. [7]	1985–1989	134 T2-3		89; 40 BCS = RT, 49 Mtx	44	–	–	22.5 BCS + RT, 22.4 after Mtx	34
Ring et al. [13]	1986–1999	453	136	67	69	74	76	10	21
Daveau et al. [10]	1985–1999	1477 T2-3	165	65	100	82	91	12	23

^a^Four-year overall survival

^b^Overall survival at 39 months

*BCS* breast conserving surgery, *cCR* clinical complete remission, *LRR* locoregional recurrence, *Mtx* mastectomy, *rD* residual disease, *RT* radiotherapy

In the study by Touboul et al. [6] performed at Hôpital Tenon (Paris, France), 97 patients with locally advanced nonmetastatic and noninflammatory breast cancer were treated between 1982 and June 1990. Three different locoregional approaches were proposed, depending on the tumor response. In 37 patients (38 %) with residual tumor noted on physical examination to be >3 cm in diameter, located behind the nipple, or with multicentricity, mastectomy and axillary dissection were performed. Sixty other patients (62 %) either had breast-conserving surgery or no surgery: 33 patients (34 %) achieved complete remission and had no surgery with an additional radiation boost, and 27 patients (28 %) who had a residual mass ≤3 cm in diameter were treated by wide excision and axillary dissection followed by a boost to the excision site. The 5-year actuarial locoregional relapse rate was 16 % after radiotherapy alone, 16 % following wide excision and radiotherapy, and 5.4 % following mastectomy (*p* = 0.04). Five-year and 10-year overall survival rates were not influenced by the local treatment.

The randomized trial reported by Mauriac et al. [7] from Institut Bergonié (Bordeaux, France) included 272 women with operable breast adenocarcinoma >3 cm. Of these, 124 were treated by initial chemotherapy. Forty-four patients had a complete clinical remission and were treated with radiotherapy only. Forty patients with residual tumor (<20 mm) were treated with lumpectomy, axillary node dissection, and radiotherapy. Forty-nine patients with residual tumors (>20 mm) had mastectomies. After a median follow-up of 34 months there was a slight increase in locoregional recurrence in the group of complete responders to primary chemotherapy that did not have surgery (four of 44) compared with those partial responders who did (two of 40).

In the study by Scholl et al. [8] from Institut Curie (Paris, France), 45 patients achieved complete clinical response after NCT and underwent radiotherapy alone. Their 5-year local recurrence-free survival was significantly lower than that in the group of 23 patients with partial response who underwent surgery when there was residual clinical disease after the first 54 Gy delivered locoregionally (70 % vs. 84 %). Similar findings have been reported in the prospective study by Perloff et al. [9] from the Cancer and Leukemia Group B and in several retrospective series exploring the potential of omitting surgery (Table 1) [8, 10–13].

In a more recent large retrospective series by Daveau et al. [10] from Institut Curie involving patients treated between 1985 and 1999 with NCT, no significant differences in overall, disease-free, and metastasis-free survival rates could be demonstrated between the surgical group (*n* = 65) and the exclusive radiotherapy group (*n* =100). However, higher locoregional recurrence rates were observed in the no-surgery group (31 % vs. 17 % at 10 years; *p* = 0.06). The prospective study by De Lena et al. [11] showed no significant difference between the treatment groups with regard to local failure (29.6 % vs. 31.1 %). Notwithstanding, local failure rates in the 30 % range would be considered unacceptable in the modern era. Mauri et al.’s [14] meta-analysis demonstrated significant increased relative risk for locoregional recurrences when radiotherapy without surgery was adopted.

In a more recent retrospective study of core biopsy alone of the breast primary followed by radiotherapy, Clouth et al. [15] reported a local recurrence of 13 % (two of 16) at a mean of 33.5 months in patients with pCR. The main issue with this study was that the multiple negative core needle biopsies were performed by random nonimage-guided biopsy of the quadrant of the breast where the tumor was and under the nipple at the time of surgery for the axillary lymph nodes (ALNs). These results underscore therapeutic strategies reflecting a previous era and the essential need to utilize state-of-the-art breast imaging with biopsy.

## Breast and nodal imaging for response monitoring and prediction of pCR

There has been increasing interest in determining whether negative imaging after systemic therapy might identify a subset of patients that could be treated safely with radiation alone without surgery. Safe omission of surgery in patients who receive neoadjuvant therapy and achieve radiologic complete response (rCR) depends on the ability to accurately estimate pCR preoperatively. However, it is important to keep in mind that even with the best imaging of the breast and nodal regions, sufficient sensitivity and specificity to select patients who indeed have no residual disease is currently lacking. Further, overall complete radiologic response may occur in a very small minority of patients in the 20 % range [16], and despite this up to 50–60 % of patients will indeed have no residual disease among patients with TN and HER2Neu-positive breast cancers. Also, there is variation in the definition and assessment methods of a clinical or imaging complete response among studies despite the standard criteria, since they fail to address breast imaging-specific aspects in detail.

The majority of previous studies have evaluated the absolute measurement of residual disease by several imaging modalities and not the attainment of pCR. To further specify and quantify pCR prediction via breast imaging (magnetic resonance imaging (MRI), mammography, ultrasound) several studies have been performed to assess the false negative rate (FNR) and the negative predictive value (NPV), which are expected to be the most important and interpretable measures for addressing the question of diagnostic prediction (Table 2). The FNR quantifies the number of patients with residual tumor not detected. The NPV quantifies the number of patients correctly identified as pathologic complete responders. The accuracy varied greatly, both among the different imaging modalities and for the breast and axilla, which demonstrates that at present there is no optimal imaging modality for pCR.

**Table 2:** False-negative rates and negative predictive values for predicting pathologic complete response in mammography, magnetic resonance imaging, and ultrasound

Study	Mammography		Ultrasound		Magnetic resonance imaging		PET/CT	
	NPV (%)	FNR (%)	NPV (%)	FNR (%)	NPV (%)	FNR (%)	NPV (%)	FNR (%)
**Breast**								
Schott et al. [19]	91	9	91	9	94	6	–	–
Peintinger et al. [18]	NPV 93, FNR 7				–	–	–	–
Chen et al. [38]	–	–	–	–	74	26	–	–
Bhattacharyya et al. [39]	–	–	–	–	96	–	–	–
Keune et al. [17]	86	–	85	–	–	–	–	–
Croshaw et al. [20]	30	70	33	67	44	56	–	–
De Los Santos et al. [16]	–	–	–	–	47^a^	–	–	–
Schaefgen et al. [21]	52	13	51	24	60	4	–	–
**Axilla**								
Kuerer et al. [40]	–	–	44	–	–	–	–	–
Vlastos et al. [41]	–	–	49	–	–	–	–	–
Klauber-Demore et al. [42]	–	–	52	48	–	–	–	–
Hsiang et al. [43]					78	38	–	–
Javid et al. [29]	–	–	–	–	81	19		–
Rousseau et al. [44]	–	–	29	–	–	–	95	–
Hieken et al. [28]	–	–	57	–	43	–	61	–
Koolen et al. [45]	–	–	–	–	–	–	73	27
Boughey et al. [31]	–	–	–	9.8^b^	–	–	–	–

^a^NPV increased to 60 % among triple-negative cases and 62 % among hormone receptor-negative HER2-positive cases

^b^Overall, 39.0 % of patients had pathologic negative nodes at axillary dissection, yet 70.4 % of axillary ultrasound images were classified as normal, suggesting that ultrasound lacks specificity to determine a pathologic complete nodal response. The FNR rate of sentinel lymph node biopsy based on ultrasound findings after chemotherapy was not significantly different. However, if only patients with normal axillary ultrasound images were selected, the FNR would drop from 12.6 % to 9.8 % for sentinel node biopsy

*CT* computed tomography, *FNR* false negative rate, *NPV* negative predictive value, *PET* positron emission tomography

### Conventional imaging

Mammography and breast ultrasonography (US) are the most commonly used imaging modalities for estimating primary tumor size at the initial diagnosis. However, their accuracy for assessing residual tumor and tumor changes following NCT is variable given the development of tumor fibrosis, fragmentation, remaining intraductal carcinoma after the disappearance of the invasive component, and/or change in tumor density.

In a retrospective review of 192 patients no difference was found in the ability of mammography or breast ultrasound to predict pCR [17]. However, when both mammography and breast ultrasound demonstrated no residual disease the likelihood of pCR was 80 %, which is similar to results recently reported by Peintinger et al. [18] from The University of Texas MD Anderson Cancer Center (Houston, TX, USA). The use of both imaging modalities improved the accuracy of predicting pCR to NCT in a greater percentage of cases than use of either modality alone. In several contemporary reports, neither mammogram or ultrasound (and MRI) is able to predict pCR (ypT0) with sufficient accuracy to replace the pathologic diagnosis of a surgical excision specimen [19–21].

### Microcalcifications and predicting residual ductal carcinoma in situ and invasive carcinoma

The presence of residual ductal carcinoma in situ does not affect long-term outcome, but has clinical implications regarding the surgical management of the patient and may at times lead to the need for more extensive resections, including the need for mastectomy despite excellent response of the invasive component to NCT. The intraductal component of a tumor can also be eradicated with NCT. Several reports exist on the effect of NCT on microcalcifications (Table 3). Microcalcifications can increase, decrease, or remain stable after the administration of NCT. However, the majority of these reports demonstrate no changes in the malignant appearing microcalcifications after NCT. In theory, if there is no documented residual carcinoma and only microcalcifications, the microcalcifications might be able to be followed and biopsied at a later time if in fact there were increases or morphologic suspicious changes.

**Table 3:** Overview of studies reporting on neoadjuvant chemotherapy and microcalcifications

Study	Number of patients and results
Matsuo et al. [46]	Strong correlation in pCR between the invasive and noninvasive components
Reports on the effect of NAC on microcalcifications	
Moskovic et al. [47]	Residual microcalcifications because of calcification of necrotic material remaining from the tumor or even fat necrosis or hematoma formation after biopsy
Vinnicombe et al. [48]	The persistence of calcifications does not necessarily indicate the presence of ductal carcinoma in situ
Fadul et al. [49]	Among patients who developed microcalcifications during NAC, they were histologically associated with both intraductal and invasive carcinomas
Adrada et al. [50]	No correlation between change in the extent of calcifications before and after neoadjuvant and pCR. Extent of calcifications on mammography following NAC does not correlate with the extent of residual disease in up to 22 % of women

*NAC* neoadjuvant chemotherapy, *pCR* pathologic complete response

### Functional imaging

Metabolic and vascular-related changes with NCT cannot be assessed by conventional methods, but functional imaging techniques such as dynamic contrast-enhanced MRI, diffusion-weighted imaging, and nuclear imaging have been used with more success. Contrast-enhanced MRI depicts changes in morphology and perfusion, whereas positron emission tomography (PET)/computed tomography (CT) visualizes changes in glucose metabolism.

## Magnetic resonance imaging

The accuracy of MRI in estimating postchemotherapy residual disease varies with tumor subtype. Accuracy is highest in estrogen receptor (ER)-negative/HER2-positive and TN tumors and lowest in luminal tumors.

A meta-analysis by Marinovich et al. [22] included 44 studies between 1990 and 2008 with, in total, 2050 patients undergoing MRI and/or comparator tests to evaluate residual disease after NCT. Studies generally showed high sensitivity (correct detection of residual tumor), with evidence of heterogeneity in the estimates of specificity (correct identification of pCR). The capability of MRI for differentiating the presence of residual malignancy from pCR had an overall area under the curve of 0.88 and this overall accuracy differed according to the definition of pCR and the study timeframe. Similarly, MRI has been observed to have limitations in detecting scattered, microscopic tumor foci after NCT [23, 24].

As demonstrated in a meta-analysis by Houssami et al. [2], different breast cancer subtypes show different pCR rates. Loo et al. [25] found that the changes in MRI during NCT correlate well with pathology outcome in TN and HER2-enriched tumors, but not in ER-positive/HER2-negative tumors. Data from the multicenter De Los Santos et al. [16] study among 746 patients showed that only 24 % achieved rCR, and overall 24 % achieved pCR. The NPV was highest for patients who had HR-negative/HER2-positive and TN breast cancers but only in the 60 % range. They confirmed that, among patients who achieved rCR, positive HR status and low tumor grade were most commonly associated with residual disease at surgery, suggesting that rCR on preoperative MRI in these patient populations should be interpreted with caution.

## Positron emission tomography

PET imaging early during chemotherapy may help tailor treatment by identifying early nonresponders given its pooled sensitivity of 84 % (range 78–88 %) and pooled specificity of 66 % (range 62–70 %) [26]. The main limitations of PET and PET/CT for evaluating primary breast lesions are their inability to detect lesions measuring <1 cm reliably and to differentiate benign from malignant pathology, resulting in a relatively high false positive rate. Accuracy of PET/CT in prediction of pCR appears to be subtype dependent and highest among ER and TN tumors.

## Predicting nodal response and axillary imaging

As trials commence evaluating the safety of eliminating surgery in the breast after NCT, surgical management of the axillary nodes becomes an issue to be considered as well. Ultrasound imaging is a fast, noninvasive, and inexpensive modality to evaluate the axilla. The presence of cortical thickening is the main feature used to determine malignant ALNs. In a meta-analysis by Alvarez et al. [27] the accuracy of preoperative US to detect ALN metastases was evaluated. The gold standard in the nine included studies was either ALN dissection or sentinel node biopsy (SNB). In studies that used lymph node size as the criterion for positive ALN, the overall sensitivity and specificity were 68 % (range, 66–73 %) and 88 % (range, 44–97 %), respectively. The overall sensitivity and specificity were 82 % (range, 68–92 %) and 96 % (range, 80–97 %), respectively [27]. Ultrasound-guided biopsy of the sonographically suspicious nodes increases the specificity to 100 %. Negative ultrasound results do not exclude ALN metastases.

Hieken et al. [28] retrospectively analyzed the performance of axillary US, MRI, and fluorine-18 fluorodeoxyglucose (FDG)-PET in detecting ypN-positive disease after NCT. In their study, 128 of 272 patients had ypN-positive disease. Post-NCT imaging included axillary US (146 patients), MRI (139 patients), and FDG-PET (38 patients). Axillary US was the most sensitive test for detecting ypN-positive disease after NCT, with a sensitivity of 70 % compared with FDG-PET (63 %) and MRI (61 %). The accuracy was best for FDG-PET (72 %) followed by axillary US (65 %) and MRI (60 %). The NPVs of axillary US, MRI, and FDG-PET were 56.8 %, 42.5 % and 61.1 %, respectively. The accuracy of current imaging modalities in predicting the axillary nodal response to treatment was 60–72 %. Therefore, imaging does not necessarily preclude surgical axillary staging for patients after completion of NCT [28].

In a prior report of 47 cN-positive patients (as determined by SNB or image-guided axillary node biopsy) on the performance of post-NCT breast MRI, the sensitivity and specificity of MRI to identify residual pathologic ALN disease following NCT were 85.7 % and 89 %, respectively, while the positive predictive value and NPV were 92 % and 80.9 %, respectively [29]. In the node-positive patients MRI was able to predict with moderate sensitivity and specificity whether residual nodal disease was present. The sensitivity of MRI is not yet sufficient to replace the gold standard of pathologic or cytologic examination for diagnosis of axillary node metastases. The accuracy of MRI is not adequate to obviate either the need for staging with SNB or the need for completion axillary dissection in women determined to be node-positive prior to neoadjuvant therapy.

The reported accuracy of FDG-PET for detecting ALN metastases varies from 74 to 95 % with a sensitivity of 61–95 % and a NPV from 79 to 95 % [30].

## Limiting and potential avoidance of surgery for node-negative and node-positive disease after NCT

pCR in the axilla is achieved in 40–75 % of patients with clinically occult or biopsy-proven positive lymph nodes [5, 31]. Restaging the axilla with ultrasound, MRI, and PET has thus far been inadequate in predicting pathologic response. Several studies have been performed to evaluate the safety of sentinel lymph node (SLN) biopsy in node-positive patients after NCT. In the ACOSOG Z1071 trial [31] the overall nodal conversion rate was 41.1 % and was dependent upon the receptor subtype. pCR was achieved in 21.1 % of patients with HR-positive disease, in 49.4 % of patients with TN disease, and in 64.7 % of those with HER2-positive disease. The FNR of SLN after NCT was 12.6 % when at least two sentinel nodes were excised, with an improving FNR when dual tracers (FNR 10.8 %) were used and when three or more nodes were removed (FNR 9.1 %) [31]. The SENTINA study and the Canadian SN FNAC study showed similar results [32, 33]. Whether these patients with undetected residual axillary disease are at increased risk of recurrence remains unclear. The advantage of performing SLN biopsy after NCT is that it reveals the nodal status after NCT, which is a better prognostic indicator than the identification of occult metastases before NCT. On the other hand, radiotherapy may be sufficient for local regional control of the axilla among patients who are clinically node negative before and after NCT and in whom there is pCR in the primary site. The need for axillary node surgery (SLN and or axillary node dissection if metastatic disease) in this group of patients can be tested.

Several trials have been developed under the concept that the best lymph node to evaluate after chemotherapy in order to determine response is the node that had confirmed metastases before therapy (the biopsied node). The hypothesis tested in these trials is that pathological changes in the involved node pre-chemotherapy accurately reflect the response to therapy in the other nodes. A prospective registry trial from MD Anderson Cancer Center demonstrated that placing a clip in the biopsy-proven positive lymph node reduced the FNR compared with SLN biopsy alone [34]. To evaluate whether a clipped node could be selectively localized and removed, a new procedure called targeted axillary dissection (TAD) was developed [34]. A ^125^I seed is placed in the clipped node 1–5 days before surgery, followed by radioisotope injection preoperatively or intraoperatively. A gamma probe is used to remove the clipped node with the seed and any other remaining sentinel nodes. The Netherlands Cancer Institute (Amsterdam, the Netherlands) introduced the marking ALNs with radioactive iodine seeds (MARI) procedure to evaluate axillary response during NCT [35]. A radioactive seed is placed at the time of the initial biopsy if metastases are confirmed and left in place through NCT. At the time of surgery, the marked node is retrieved with a gamma probe and examined. The radioactive node was identified in 97 % of the patients. ALN dissection showed a FNR of 7 %. pCR was obtained in 26 % of patients.

If percutaneous biopsy can be utilized at the beginning of therapy to document axillary nodal metastases, why not do the same thing following chemotherapy? This hypothesis is currently being examined in an MD Anderson Cancer Center study which correlates fine needle aspiration with surgical excision to assess for eradication of nodal metastases after NCT in breast cancer by biopsy of the same node that harbored carcinoma prior to NCT [36]. If the results prove accurate, patients who converted from documented nodal disease to axillary pCR could receive just radiotherapy alone.

## Current trials evaluating the potential for eliminating breast cancer surgery after NCT

More recently, several groups have generated potential concept trials to further investigate the possibility of avoiding surgery after NCT [16, 37]. MD Anderson Cancer Center recently opened and has begun accrual on the trial ‘Identification of breast cancer patients for potential avoidance of surgery: Accuracy of image guided percutaneous sampling compared with surgery to evaluate eradication of breast cancer after preoperative chemotherapy’ (Clinicaltrials.gov NCT02455791). The study is designed to evaluate whether patients with residual carcinoma can be accurately identified using state-of-the-art image-guided fine needle aspiration and vacuum-assisted core needle biopsies. One major difference in the ongoing MD Anderson Cancer Center study is that patients are not required to have a complete radiologic response and only patients who have TN disease and HER2-positive disease are eligible. This molecular subtype was chosen because a complete pathologic response is very common with systemic therapy. Additionally, as the imaging response is often inaccurate in these clinical subtypes, the addition of multiple different types of biopsies is designed to identify patients without residual disease despite apparent residual disease on breast imaging. The accuracy of this intervention will be documented with routine surgery and complete pathologic evaluation. This trial serves as the foundation for the subsequent planned MD Anderson Cancer Center study to ensure that patients can safely be identified for the pivotal exceptional responder treatment trial which will avoid surgery completely.

The Netherlands Cancer Institute as well as other national and international single-center and multicenter and cooperative groups are developing similar trials. Patients with TN disease and HER2-positive disease with complete pathologic response following NCT are known to have reduced local regional and distant recurrences compared with patients with remaining disease [3]. Long-term follow-up on subsequent treatment trials will be needed to evaluate local recurrence, disease-free survival, and overall survival.

## Conclusions

pCR rates are highly improving, especially in TN and HER2-positive subgroups. Given these high response rates in defined subgroups it is appropriate to question whether surgery is now a redundant procedure in their management. Safe omission of surgery in patients who receive neoadjuvant therapy is dependent on the ability to accurately estimate pCR preoperatively and current imaging is lacking sufficient sensitivity and specificity to select patients who indeed have no or only very limited residual disease. Further studies are needed to determine the best clinical assessment of pathological tumor response to NCT. Landmark trials using state-of-the-art image-guided biopsy after NCT to test the safety of potential avoidance of breast cancer surgery in exceptional responders are under development and foundation studies are underway.

## Abbreviations

ALN: axillary lymph node
CT: computed tomography
ER: estrogen receptor
FDG: fluorine-18 fluorodeoxyglucose
FNR: false negative rate
HER2: human epidermal growth factor receptor 2
HR: hormone receptor
MARI: marking axillary lymph nodes with radioactive iodine seeds
MRI: magnetic resonance imaging
NCT: neoadjuvant chemotherapy
NPV: negative predictive value
pCR: pathologic complete response
PET: positron emission tomography
rCR: radiologic complete response
SLN: sentinel lymph node
SNB: sentinel node biopsy
TAD: targeted axillary dissection
TN: triple negative
US: ultrasonography
